# Right hemisphere brain lateralization for knee proprioception among right-limb dominant individuals

**DOI:** 10.3389/fnhum.2023.969101

**Published:** 2023-01-19

**Authors:** Andrew Strong, Helena Grip, Ashokan Arumugam, Carl-Johan Boraxbekk, Jonas Selling, Charlotte K. Häger

**Affiliations:** ^1^Department of Community Medicine and Rehabilitation, Physiotherapy, Umeå University, Umeå, Sweden; ^2^Department of Radiation Sciences, Umeå University, Umeå, Sweden; ^3^Department of Physiotherapy, College of Health Sciences, University of Sharjah, Sharjah, United Arab Emirates; ^4^Danish Research Centre for Magnetic Resonance, Centre for Functional and Diagnostic Imaging and Research, Copenhagen University Hospital Hvidovre, Copenhagen, Denmark; ^5^Umeå Center for Functional Brain Imaging, Umeå University, Umeå, Sweden; ^6^Institute of Sports Medicine Copenhagen and Department of Neurology, Copenhagen University Hospital Bispebjerg, Copenhagen, Denmark; ^7^Institute for Clinical Medicine, Faculty of Medical and Health Sciences, University of Copenhagen, Copenhagen, Denmark

**Keywords:** proprioception, functional magnetic resonance imaging (fMRI), brain, motion capture, functional laterality, lower extremities, knee, motor control

## Abstract

**Introduction:**

Studies indicate that brain response during proprioceptive tasks predominates in the right hemisphere. A right hemisphere lateralization for proprioception may help to explain findings that right-limb dominant individuals perform position matching tasks better with the non-dominant left side. Evidence for proprioception-related brain response and side preference is, however, limited and based mainly on studies of the upper limbs. Establishing brain response associated with proprioceptive acuity for the lower limbs in asymptomatic individuals could be useful for understanding the influence of neurological pathologies on proprioception and locomotion.

**Methods:**

We assessed brain response during an active unilateral knee joint position sense (JPS) test for both legs of 19 right-limb dominant asymptomatic individuals (females/males = 12/7; mean ± SD age = 27.1 ± 4.6 years). Functional magnetic resonance imaging (fMRI) mapped brain response and simultaneous motion capture provided real-time instructions based on kinematics, accurate JPS errors and facilitated extraction of only relevant brain images.

**Results:**

Significantly greater absolute (but not constant nor variable) errors were seen for the dominant right knee (5.22° ± 2.02°) compared with the non-dominant left knee (4.39° ± 1.79°) (*P* = 0.02). When limbs were pooled for analysis, significantly greater responses were observed mainly in the right hemisphere for, e.g., the precentral gyrus and insula compared with a similar movement without position matching. Significant response was also observed in the left hemisphere for the inferior frontal gyrus pars triangularis. When limbs were assessed independently, common response was observed in the right precentral gyrus and superior frontal gyrus. For the right leg, additional response was found in the right middle frontal gyrus. For the left leg, additional response was observed in the right rolandic operculum. Significant positive correlations were found between mean JPS absolute errors for the right knee and simultaneous brain response in the right supramarginal gyrus (*r* = 0.464, *P* = 0.040).

**Discussion:**

Our findings support a general right brain hemisphere lateralization for proprioception (knee JPS) of the lower limbs regardless of which limb is active. Better proprioceptive acuity for the non-dominant left compared with the dominant right knee indicates that right hemisphere lateralization may have meaningful implications for motor control.

## Introduction

In 1906, Sherrington first used the term proprioception to describe how the body acts as a stimulus to its own receptors (Sherrington, 1906). Proprioception was recently described by Heroux et al. (2022) as “the awareness of the mechanical and spatial state of the body and its musculoskeletal parts.” Specifically, proprioception encompasses the senses of position, movement, force and effort (Proske and Gandevia, 2012). These proprioceptive senses depend upon feedback signals transmitted to the central nervous system (CNS) from mechanosensory neurons distributed throughout the body called proprioceptors (Tuthill and Azim, 2018). Afferent feedback from the proprioceptors is processed in regions of the brain such as the primary somatosensory cortex, supramarginal gyrus and insula (Chilvers et al., 2021). Our understanding of the brain regions involved in the processing of proprioceptive signaling and the lateralization of such activity is, however, still at an early stage (Tuthill and Azim, 2018).

Each limb is primarily controlled by brain regions of the contralateral side (Vulliemoz et al., 2005). Despite this, evidence indicates a lateralization of brain activity for proprioceptive tasks, irrespective of limb dominance, whereby the right hemisphere appears to be predominant (Naito et al., 2005; Goble et al., 2011; Ben-Shabat et al., 2015; Iandolo et al., 2018; Chilvers et al., 2021; Strong et al., 2022b). The supporting evidence for proprioception-related brain laterality is, however, limited in quantity and largely restricted to the upper limbs. We recently reported a general lateralization of brain response in the right hemisphere during a knee joint position sense (JPS) test among a pooled group of asymptomatic individuals and persons with anterior cruciate ligament reconstruction (ACLR) (Strong et al., 2022b). A right hemisphere lateralization may help to explain why right-limb dominant individuals have been shown in several studies to perform significantly better at position matching tasks with the non-dominant left-side joints such as the shoulder (Han et al., 2013), elbow (Kurian et al., 1989; Goble et al., 2006; Goble and Brown, 2007, 2008), thumb (Roy and MacKenzie, 1978; Nishizawa, 1991), and fingers (Han et al., 2013) compared with the corresponding dominant right-side joints. For the lower limbs, some evidence for a preference of the non-dominant side for position matching exists for the knee (Han et al., 2013), ankle (Symes et al., 2010; Han et al., 2013), and foot (Iandolo et al., 2018). Contradictory findings for a lack of influence for side dominance on lower limb position matching (Bullock-Saxton et al., 2001; Galamb et al., 2018) nevertheless cause uncertainty in this area for the lower extremities.

A better understanding of the neural correlates of proprioception for the lower limbs could provide valuable information relating to motor control. Studies combining brain imaging and quantifiable lower limb proprioception are, however, scarce. One recent study did find better position matching of the non-dominant left foot and brain activation predominantly in the right parietal and frontal cortex for both ipsilateral and contralateral position matching regardless of which foot (right dominant or left non-dominant) was active among asymptomatic individuals (Iandolo et al., 2018). Our own recent study of knee JPS (Strong et al., 2022b) included asymptomatic participants and those with unilateral ACLR of either knee, thus complicating side-to-side comparisons. Significant positive correlations were nevertheless found between knee JPS errors and response in the right anterior cingulate and supramarginal gyrus when the right leg was active, as well as response in the left insula when the left leg was active. A separate between-leg comparison of knee JPS outcomes and simultaneous brain response among only the asymptomatic controls of our study provides an opportunity to shed light on the influence of dominance on knee proprioception and lateralization of related brain response.

The aim of this study was thus to investigate the influence of side dominance of the lower limbs on knee JPS and the lateralization of related brain response among asymptomatic right-limb dominant individuals. It was hypothesized that participants would show significantly better knee proprioception for the non-dominant left side compared with the dominant right side. It was further hypothesized that brain response, regardless of which leg was active, would show predominantly right hemisphere activation in, e.g., somatosensory cortices and insula.

## Materials and methods

### Participants

This study had a cross-sectional design. Participants were recruited between August 2017 and May 2019 using convenience sampling via sports clubs, advertisements at the local University, social media, and word of mouth. Screening ensured that participants met the following initial eligibility criteria: aged 17–35 years, magnetic resonance imaging compliance, right-limb dominance (foot preferred to kick a ball and hand used for writing), ability to understand either Swedish or English language, and no known previous or ongoing injuries or diseases that could affect the CNS or leg movements. Of the 61 individuals who showed an interest in participating as an asymptomatic control, 47 met the initial eligibility criteria. The participants of this study also formed a control group for comparisons with an ACLR group in a previous study (Strong et al., 2022b) and were thus further screened in order to match the ACLR group with regard to sex distribution, body height, body mass, and activity level. Thus, 20 of the individuals who met the initial eligibility criteria were invited to participate and completed testing. During imaging analyses, one participant was however found to have a benign arachnoid cyst and was subsequently excluded to prevent confounding of analyses. Therefore, 19 asymptomatic right-limb dominant (both hand and foot) individuals (12 females) were included in the final analyses of this study. See Table 1 for the characteristics of the study group. The project was approved by the Regional Ethical Review Board in Umeå, Sweden (Dnr. 2015/67-31) and was conducted in accordance with the Declaration of Helsinki. Participants provided their written informed consent prior to participation.

**TABLE 1:** Participant characteristics of the study group (*n* = 19).

Age, years	27.1 (4.6)
Male:female, n	7:12
Body height, m	1.75 (0.08)
Body mass, kg	73.1 (9.9)
**Patient-reported outcomes, median (range)**	
Marx activity score*	11.0 (7.0)
Tegner activity score**	6.0 (4.0)

Mean (SD) unless otherwise stated.

*Marx scale minimum-maximum 0–16.

**Tegner scale minimum-maximum 0–10.

### Procedures

All participants completed the Marx Activity Scale (Marx et al., 2001) and the Tegner Activity Scale (Tegner and Lysholm, 1985) to provide knee-specific physical activity level, as this has previously been shown to influence knee JPS outcomes (Strong et al., 2021). The Marx Activity Scale reports the frequency of running, cutting, deceleration and pivoting actions performed during the previous year when the individual was most active (scale of 0–4 for each four actions: 0 = <1×/month; 1 = 1×/month; 2 = 1×/week; 3 = 2–3×/week; 4 = ≥ 4x/week [minimum-maximum combined score = 0–16]). The Tegner Activity Scale was designed for individuals with ACL injury and reports the highest level of possible activity based on occupational and sporting activities (scale of 0–10). For familiarization purposes, participants first performed a simplified version of our supine knee JPS test in the U-motion laboratory at Umeå University, Sweden. An extended, fMRI-adapted protocol of the knee JPS test was then performed approximately one hour later in an MRI scanner at the Umeå center for Functional Brain Imaging, University Hospital of Umeå, Sweden.

### Knee joint position sense test protocol

The knee JPS test protocol has been described in detail in our previous study (Strong et al., 2022b). Briefly, participants lay in a supine position in the MRI scanner with their feet and shanks strapped to foot holders of a custom-made low-friction knee flexion/extension board, which permitted movements similar to a heel slide exercise. A strap secured the torso and cushions in the head coil were used to reduce head movements. An angled mirror attached to the head coil enabled sight of instructions which appeared on a screen at the back of the scanner. Test instructions were activated based on knee angles and angular velocities registered by a three-camera motion capture system (Oqus MRI Qualisys AB, Gothenburg, Sweden, 120 Hz). Knee angles were calculated based on the positions of passive retro-reflective markers which were affixed to participants at the greater trochanter and lateral epicondyle of each femur, as well as on the lateral side of each foot holder of the sliding board in line with each lateral malleolus. Participants practiced a knee angular velocity of 10°/s during familiarization and were requested to adhere to this velocity for all movements. Test-retest reliability for the simplified version of the JPS test when performed by a separate group of 15 (9 females) asymptomatic persons in a motion analysis laboratory has been reported previously (Strong et al., 2022b) for the non-dominant (ICC 3,10 = 0.64 [CI 0.02–0.87], SEM = 0.67°) and dominant leg (ICC 3,10 = 0.78 [CI 0.34–0.93], SEM = 0.86°).

The protocol involved three experimental conditions: (1) *JPS* condition: participants flexed one leg until a stop sign appeared on the screen (activated at either 35° or 60° knee flexion). This knee (target) angle was instructed to be memorized and after 8 s participants returned their leg to the start position. Participants then attempted to reproduce the target (memorized) knee angle with the same leg. Eight repetitions of the *JPS* condition were performed for each angle and leg, resulting in 16 repetitions per leg. Brain images were extracted for analyses during the entire reproduction phase, i.e., from onset of flexion to cessation of flexion when reproducing the target angle. (2) *Flex* condition: simple knee flexion from full extension to approximately 100° and back to full extension. Eight repetitions of the *Flex* condition were performed per leg. Brain images were extracted for analyses from onset of flexion to 65° knee flexion angle. (3) *Rest* condition: lying still in the start position for 15 seconds. Five repetitions of the *Rest* condition were performed. Brain images were extracted for the entire duration of the condition. The *JPS* and *Flex* conditions were performed in a pseudorandomized order to ensure a maximum of two consecutive repetitions of any condition and at least 7 s between trials. The *Rest* condition was included at evenly-spaced intervals throughout the protocol. The entire protocol lasted approximately 40 minutes and resulted in 1,240 whole-brain sets.

### Image acquisition

Structural and functional brain images were acquired using a 3T General Electric MR scanner with a 32-channel head coil. To create a study-specific template, a T1 structural image was first acquired using the following parameters: 180 slices; 1 mm thickness; repetition time 8.2 ms; echo time 3.2 ms; flip angle 12°; field of view 25 cm × 25 cm. Collection of the functional gradient-echo-planar imaging sequence was performed with the following scanning parameters: repetition time = 2,000 ms, echo time = 30 ms, flip angle = 80°, field of view = 25 cm × 25 cm. Thirty-seven transaxial slices were acquired in an interleaved order with a thickness of 3.4 mm (0.5 mm gap). Ten initial dummy scans were collected and discarded prior to analysis. A tilted mirror attached to the head coil enabled sight of test instructions which were presented on a computer screen. To synchronize kinematic data with fMRI data for later analyses, the computer parallel port was used to detect the trigger output signal from the MR scanner.

### Data processing and analysis

Kinematic data were filtered with a 6 Hz fourth-order low-pass zero-lag Butterworth filter using the Visual3D software (v.5.02.19, C-Motion Inc. Germantown, MD, USA). Automated scripts were used to set events based on sagittal plane knee kinematics (movement angles and velocities). Events were visually inspected by the lead researcher (AS), but none were adjusted, and no data were removed from analyses. Due to slow performance of the test, one included participant performed 15 rather than 16 repetitions of the *JPS* condition per leg. Outcome variables for the knee JPS test were: (i) constant error (CE): the difference between the target angle and reproduction angles of each separate trial considering the direction of error, (ii) absolute error (AE): the absolute difference between the target and reproduction angles of each separate trial, and (iii) variable error (VE): an estimate of consistency between trials based on the reproduction angle using the formula:


(1) $$V\,E=\sqrt{\left[\Sigma \,{\left(X_{i}-M\right)}^{2}/N\right]}$$

where √ = the square root, Σ = the “sum of”, X_*i*_ = score of the *i*th trial, M = the mean reproduction angle, and N = the number of trials (Schmidt and Lee, 2013).

Mean CE, AE, and VE for each participant were calculated for each leg by pooling the 40° and 65° conditions.

Automated batching, pre-processing and data analyses were performed using the SPM12 software (Wellcome Department of Cognitive Neurology, London, UK) integrated with MATLAB R 2016 b (MathWorks, Inc., Natick, MA, USA). Visualization of statistical maps was conducted using SPM and calculation of the percentage of BOLD signal change was done using MarsBaR 0.44 (Brett et al., 2002). Pre-processing of data was performed as follows: slice timing correction (interleaved order, first image set to reference slice), movement correction by unwarping and realigning all subsequent scans to the first image, co-registration of the mean functional image set and the structural T1 image set, segmentation of the co-registered structural image, normalization to a sample-specific template based on white and gray matter segments from the segmented, co-registered, structural image [using DARTEL (Ashburner, 2007)], and affine alignment to Montreal Neurological Institute (MNI) standard space and smoothing with an 8-mm FWHM Gaussian kernel. The final voxel size was 2 mm × 2 mm × 2 mm.

### Statistical analyses

All statistical analyses for knee JPS errors, participant characteristics and patient-reported outcomes were performed in IBM SPSS Statistics for Windows, version 25 (IBM Corp., Armonk, N.Y., USA). No outliers were found in the data sets. Normality of the group-level data was confirmed by Shapiro-Wilk tests and observation of distribution graphs. As data were skewed, mean knee JPS data were log-transformed to achieve normal distribution. The log-transformed JPS data were then compared statistically between legs using paired samples *t*-tests. The first-order analyses were set up by including the experimental conditions as regressors of interest in the general linear model, convolved with the hemodynamic response function. Six realignment parameters (head rotations and translations) were included as covariates of no interest to account for movement artifacts. The following contrasts were set up for each participant: (1) [*JPS* > *Rest*] and (2) [*Flex* > *Rest*]. Group analyses were based on a flexible factorial model design analyzing the interaction effect (leg × condition), where leg had two sides (right and left) and condition had two levels ([*JPS* > *Rest*] and [*Flex* > *Rest*]). Activation was defined as significant if the corrected family-wise error (FWE) rate was <0.05 with a voxel limit of >5. Brain regions that showed significant activation for any of these analyses were further analyzed by calculating the percentage of BOLD signal change during the *JPS* condition (i.e., the original beta) in the significant region, compared to the overall mean brain activity of the session. Statistically significantly higher or lower activation in these voxels for the JPS condition compared to the mean of the session are thus reported as positive and negative values, respectively. If mean BOLD signal percentage change for a particular region was lower during the *JPS* condition than its mean for the session (negative values) it was considered as irrelevant and was not analyzed further. The percentage of BOLD change values considered as relevant (larger than the mean for the session) were exported to SPSS where Spearman’s rho was used to analyze correlations between mean JPS errors. The strength of correlation was interpreted as negligible (*r* = 0.00-0.10), weak (*r* = 0.10-0.39), moderate (*r* = 0.40-0.69), strong (*r* = 0.70-0.89), or very strong (*r* = 0.90-1.00) (Schober et al., 2018). Significance levels were set a priori (α = 0.05).

## Results

### Knee joint position sense

**FIGURE 1:**
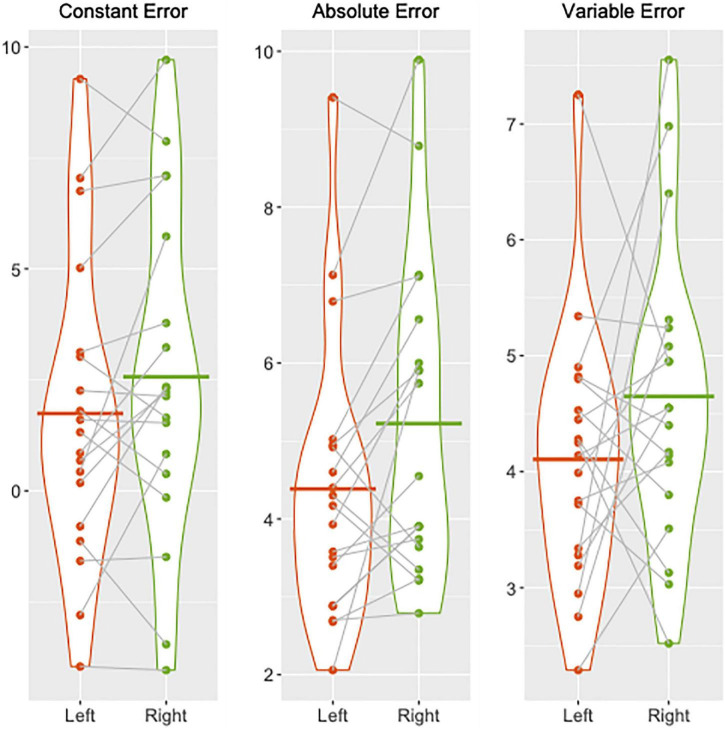
Violin plots illustrating group and individual mean knee joint position sense errors (constant, absolute, and variable) in degrees of knee flexion for the left (red) and right (green) legs. Individual means are depicted by the dots and the corresponding means for each leg are joined together by dotted lines. Group means for the left and right legs are shown by the solid red and green lines, respectively.

Participants performed the knee JPS test with significantly greater mean AE for the dominant right leg (5.22° ± 2.02°) compared with the non-dominant left leg (4.39° ± 1.79°) (*P* = 0.02). Mean CE and VE were not significantly different between legs despite the continued trend for greater mean values for the dominant leg compared with the non-dominant leg (CE: 2.57° ± 3.70° vs. 1.74° ± 3.41°, *P* = 0.10; VE: 4.65° ± 1.30° vs. 4.11° ± 1.11°, *P* = 0.18). The percentages of individuals who had smaller mean CE, AE, and VE for the left compared with the right knee were 58, 79, and 58%, respectively. Group and individual mean errors for both legs are illustrated in Figure 1.

### Brain response

**FIGURE 2:**
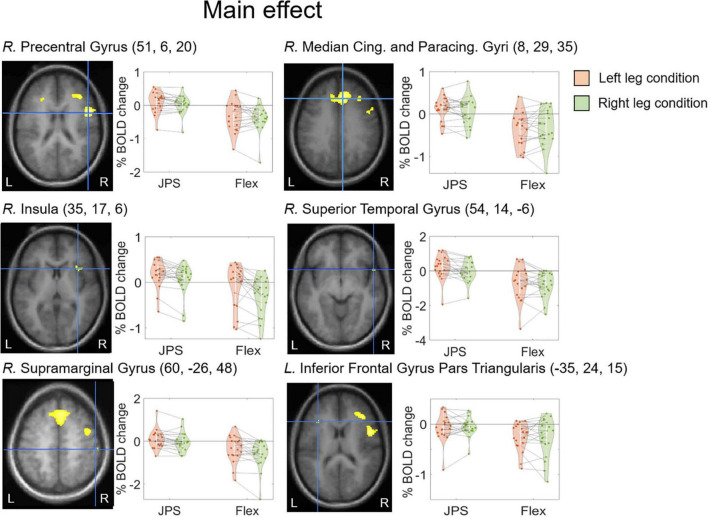
BOLD response change (%) in the center of peak activation in the brain regions with a significant main effect from condition [*JPS* > *Rest*] > [*Flex* > *Rest*], both legs included. The crosshair is centered on the peak voxel of the significant region, visualized on the group mean structural image. Beta representing *JPS* and *Flex* are illustrated for both legs (green = right leg condition, orange = left leg condition). Individual means are depicted by the dots and the corresponding means for each leg are joined together by dotted lines. The MNI coordinates corresponding to peak activation are provided in parentheses (X, Y, Z). Cing., Cingulate; L., left; *JPS*, joint position sense; Paracing., Paracingulate; R., right.

**FIGURE 3:**
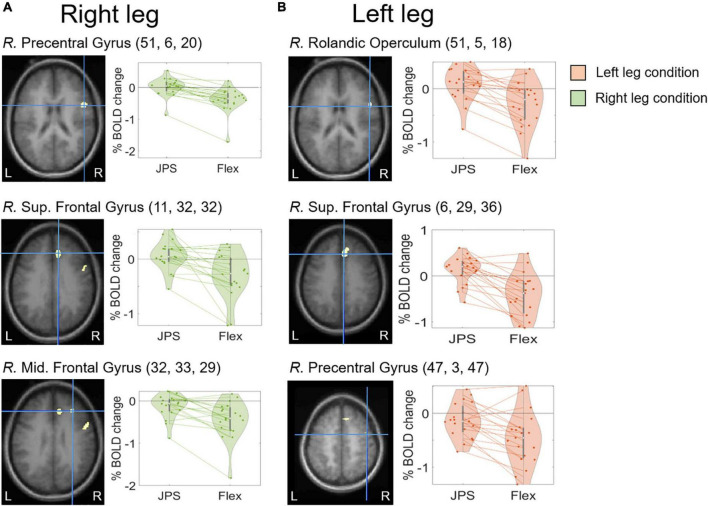
BOLD response change (%) in the center of peak activation in the brain regions with a significant main effect from [*JPS* > *Rest*] > [*Flex* > *Rest*] when analyzing right leg movement **(A)** and left leg movement **(B)** separately. The crosshair is centered on the peak activation center for each significant region, visualized on the group mean structural images. Beta representing *JPS* and *Flex* are illustrated for both legs. Individual means are depicted by the dots and the corresponding means for each leg are joined together by dotted lines. The MNI coordinates corresponding to peak activation are provided in parentheses (X, Y, Z). *JPS*, joint position sense; Mid., Middle; S, Superior; R., right.

A significant main effect for *JPS* > *Flex*, regardless of the leg tested, was seen in the right hemisphere for the precentral gyrus, median cingulate and paracingulate gyri, insula, superior temporal gyrus and supramarginal gyrus, as well as in the left hemisphere for the inferior frontal gyrus pars triangularis (see Figure 2 and Table 2 for images of all significant regions and their BOLD percentage change as well as voxel extent, exact statistics and MNI coordinates). Confirming results of our main effect analysis, the separate analyses of the right and left legs also found significant activation in the right hemisphere for the precentral gyrus and superior frontal gyrus for both legs. Additional activation was seen in the right middle frontal gyrus for the right leg and the right rolandic operculum for the left leg (see Figure 3 and Table 2 for images of all significant regions and their BOLD percentage change as well as voxel extent, exact statistics and MNI coordinates).

**TABLE 2:** Brain regions with significant main effect in condition *([JPS* > *Rest*)] > [*Flex* > *Rest*]).

Test side	Brain regions	Voxel #	*P*	Z max	MNI coordinates		
					*X*	*Y*	*Z*
Main effect	*R.* Precentral Gyrus	1475	0.000	6.04	51	6	20
	*R.* Median Cingulate and Paracingulate Gyri	4407	0.000	5.61	8	29	35
	*R.* Insula	68	0.018	4.65	35	17	6
	*R*. Superior Temporal Gyrus	12	0.020	4.63	54	14	−6
	*R*. Supramarginal Gyrus*	12	0.022	4.60	60	−26	48
	*L.* Inferior Frontal Gyrus Pars Triangularis	17	0.030	4.52	−35	24	15
Right only	*R.* Precentral Gyrus	352	0.002	5.12	51	6	20
	*R.* Superior Frontal Gyrus	185	0.008	4.83	11	32	32
	*R.* Middle Frontal Gyrus	64	0.011	4.75	32	33	29
Left only	*R.* Rolandic Operculum	84	0.006	4.90	51	5	18
	*R.* Superior Frontal Gyrus	359	0.011	4.77	6	29	36
	*R*. Precentral Gyrus	8	0.039	4.45	47	3	47

*BOLD signal percentage change was significantly correlated with JPS mean absolute error for the left leg. BOLD, blood oxygen level dependent; *JPS*, joint position sense condition; *Flex*, leg flexion without JPS task condition; *Rest*, rest condition; Test side: the leg that was active during the JPS test; Voxel #: indicates number of activated voxels in this cluster; *P*: 0.05 family wise error rate corrected (cluster level); Z max: Z-score of the voxel with the highest activity for main effect from condition; MNI: (Montreal Neurological Institute) voxel with the highest activity in MNI-space. *R*., right; *L*., left.

### Correlations between knee joint position sense and brain response

**FIGURE 4:**
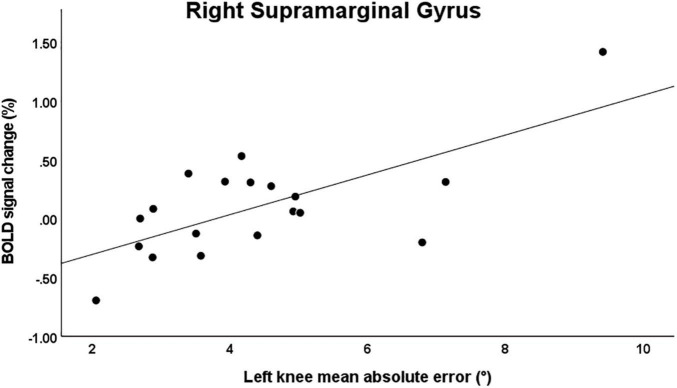
Scatter plot illustrating the significant correlation between mean knee joint position sense absolute error for the left knee and simultaneous BOLD signal percentage change in the right supramarginal gyrus (MNI coordinates [X, Y, Z]: 60, –26, 48; *r* = 0.474, *P* = 0.040). Note that when removing the two most extreme values, this correlation was no longer statistically significant.

For the main effect analysis, a significant moderate correlation was observed between knee JPS mean AE for the left side and BOLD signal change in the right supramarginal gyrus (*r* = 0.474, *P* = 0.040) (Figure 4). No other significant correlations were observed between knee JPS CE, AE or VE for either leg and BOLD response in the significantly activated brain regions during the knee JPS test. See Table 3 for all results from the correlation analyses.

**TABLE 3:** Correlations between mean knee joint position sense errors and BOLD signal change in brain regions with significant positive main effect in condition *([JPS* > *Rest*)] > [*Flex* > *Rest*]).

	Constant error		Absolute error		Variable error	
Left knee active	*r*	*P*	*r*	*P*	*r*	*P*
*R.* Precentral Gyrus	0.282	0.241	0.288	0.232	0.068	0.781
*R.* Median Cingulate and Paracingulate Gyri	0.070	0.775	0.070	0.775	0.000	1.000
*R.* Insula	0.047	0.847	0.165	0.500	0.128	0.601
*R*. Superior Temporal Gyrus	0.351	0.141	0.132	0.591	-0.032	0.898
*R*. Supramarginal Gyrus	0.172	0.482	**0**.474*	**0**.**040**	0.211	0.387
**Right knee active**						
*R.* Precentral Gyrus	0.077	0.753	0.026	0.915	0.306	0.202
*R.* Median Cingulate and Paracingulate Gyri	0.188	0.442	-0.222	0.361	-0.311	0.195

*R*., right. Significant correlations highlighted using bold text and an asterisk.

## Discussion

The main findings from this study were that asymptomatic right-limb dominant individuals performed our knee JPS test with significantly smaller absolute errors for their non-dominant left knee compared with their right knee and that regardless of which leg was reproducing the knee angles, significantly activated brain regions were predominantly located in the right hemisphere. No significant differences were however found for constant or variable errors. Our findings thus cautiously indicate a preference for the left leg during knee position matching tasks and a general right hemisphere lateralization of proprioceptive processing in the brain for individuals who prefer to kick a ball and write with the right foot and hand, respectively. These results are in agreement with previous research addressing predominantly the upper limbs and extend our knowledge to the lower limbs.

The significantly better position matching ability for the non-dominant left limb compared to the right limb is in line with evidence for a number of other body parts as detailed here previously. Some contradicting evidence has, however, found no significant difference between the dominant and non-dominant sides for the arm (Roy and MacKenzie, 1978; Naughton et al., 2002) and knee (Bullock-Saxton et al., 2001; Galamb et al., 2018). Possible reasons for the mixed results include differing methodologies regarding the many modifiable factors of the test procedures, such as different target angles and passive or active movements. For the hand, for example, differences in matching errors have been found between ipsilateral and contralateral matching protocols (Adamo and Martin, 2009). Nevertheless, support for a preference of the non-dominant side has also been found among left-hand dominant individuals for target matching tasks of the arm (Goble et al., 2009). This asymmetry for position matching in favor of the non-dominant limb is supported by Sainburg’s (2002; 2005) dynamic-dominance hypothesis, which proposes that the non-preferred side is specialized for feedback mediated control of position and the dominant side for feedforward control of trajectory features. From an applied behavioral perspective, this hypothesis can be described for the lower limbs when kicking a ball, whereby the non-dominant leg stabilizes the body as the dominant leg dynamically manipulates the target (Han et al., 2013). Right-side lateralization of brain response during position matching does however suggest that a non-dominant limb preference for proprioception may be emphasized among right-limb dominant individuals. Further research among left-limb dominant and mixed-limb dominant individuals regarding limb dominance and brain response during proprioceptive tasks is warranted.

The predominant right hemisphere brain response found among our right-limb dominant individuals is in line with a growing body of evidence for lateralization of proprioceptive processing. Chilvers et al. (2021), for example, found that deficits for arm position and movement matching were more common among individuals with brain lesions of the right hemisphere compared to those with left hemisphere lesions. Naito et al. (2005) used vibration of the extensor carpi ulnaris muscles of the hands to elicit kinesthetic illusory palmar flexion among asymptomatic individuals and found significant activation of mainly brain regions of the right hemisphere such as the anterior insula and superior temporal gyrus. In a similar study including illusory movements of the hands and feet, Naito et al. (2007) further found right hemisphere lateralization for the upper and lower limbs where both common and limb-specific sections of, e.g., motor cortices and supplementary motor area, were activated. For the lower limbs, Cignetti et al. (2014) used vibration stimulus of the right and left tendons of the tibialis anterior muscles among asymptomatic individuals and found significant activation of, e.g., right inferior frontoparietal areas. Iandolo et al. (2018) investigated ipsilateral and contralateral position matching of the foot and also found a right hemisphere dominance along with a task preference for the non-dominant left side. Specifically, activated brain regions included common areas with the current study such as the right supramarginal gyrus and primary somatosensory cortex. Our results are therefore largely in agreement with the existing research focused on the neural correlates of proprioception but extend the findings to the knee using a paradigm that incorporates a common JPS testing method.

The significant moderate, positive correlation between mean left knee JPS AE and BOLD signal percentage change for the right supramarginal gyrus indicates that worse knee JPS is correlated with greater activation in this region of the brain. It should be noted, however, that the correlation was greatly influenced by two of our participants who were found to have the most extreme values for JPS errors and BOLD response and when removing these two participants the correlation was no longer statistically significant. Nevertheless, the activated brain region does indeed appear to be of particular interest for proprioception, given findings of significant activation during, e.g., force matching at the knee (Grooms et al., 2021) and position matching at the wrist (Ben-Shabat et al., 2015). Among individuals with stroke, Chilvers et al. (2021) found correlations between worse performance of position and movement matching tasks of the arm and damage to the supramarginal gyrus, as well as the primary somatosensory cortex and superior temporal gyrus. Whether the positive correlation observed in the present study, which suggests that greater errors are associated with greater brain response, has a functional meaning is difficult to interpret. It should also be noted that this correlation is in contradiction to a previous study among a mixed group of asymptomatic individuals and persons with stroke for contralateral active wrist position matching whereby smaller errors were associated with greater response in the supramarginal gyrus (Ben-Shabat et al., 2015). The contrasting JPS testing methods between studies highlight the difficulties in collating evidence from multiple studies. For the knee, for example, differences in the sensitivity of tests may be dependent upon whether active or passive movements are used (Strong et al., 2022a) and whether unilateral or contralateral matching is performed (Hoshiba et al., 2020). More robust studies that are able to quantify proprioceptive acuity during simultaneous brain imaging are warranted, while considering the methodological challenges of estimating proprioception.

Limitations of the current study include the secondary task of trying to maintain a constant knee angular velocity. This was, however, also attempted during the contrast *Flex* condition and thus the remaining brain response can be assumed to be associated with position matching. The active movements did, however, result in variable knee angular velocities both with and between individuals. A verbal reminder to adjust the angular velocity was, however, given in instances when participants deviated consistently by 5°/s from the requested and practiced 10°/s. Mean knee angular velocity when combining knee flexion movements of both legs and each angle toward the target angles and reproduction angles was 13.48°/s ± 3.58, which shows that the participants were generally successful in maintaining a knee angular velocity within the requested range. Variations in knee angular velocity may influence the magnitude of JPS errors and are encouraged to be controlled for in future studies. A related consideration is the possibility that participants used a timing strategy to reproduce knee angles rather than using position sense of the joint. It should also be noted that although the task was specifically focused on reproducing angles of the knee, the test also involved changes in hip angle and thus contributions from both joints likely contributed to the outcomes. A multi-joint movement was considered preferential to a single-joint movement as it is more ecologically valid. Reliability of the test as performed with the current protocol in an MR scanner should also be assessed, as our existing reliability analysis was performed using a shorter protocol in a movement analysis laboratory. We also acknowledge that brain images were extracted from 16 repetitions of the JPS test per leg and that a greater number of repetitions would have allowed for a higher number of brain images to be extracted, thus increasing statistical power. The brain images that were used for analyses were however only those during which proprioceptive processing was relevant. This was possible due to simultaneous motion capture which provided integrated lower limb kinematics and thus allowed us to set specific and accurate timeframes of interest for data extraction. Our brain imaging analyses were therefore not convoluted with data unrelated to proprioception. Evidence among older adults suggests that cortical lateralization reduces with age (McGregor et al., 2009; Landelle et al., 2020). Our participants were 18–35 years of age and thus we did not expect to see an influence of age among this younger group. However, we were not able to investigate this among the current study population due to an insufficient number of participants and it is therefore unclear whether including this age range influenced our results. We nevertheless speculate based on the existing evidence that, for example, had we included participants with a lower maximum age we may have seen greater lateralization. We encourage future research to investigate whether cortical lateralization is influenced by age during proprioceptive tasks of the lower limbs. The categorization of leg dominance is also of interest and was in the current study based on asking the participants which foot they preferred to kick a ball. Leg dominance has, however, been shown to be task-specific and thus it is unclear whether using such a classification for an unfamiliar task is optimal (van Melick et al., 2017). Furthermore, determining handedness and footedness based on only single dichotomous questions for each offers less insight into the level of dominance than assessments with multiple items, e.g., the Edinburgh inventory for handedness (Oldfield, 1971). Global handedness as assessed in the current study has, however, been shown to have high agreement with laterality scores based on the Edinburgh inventory (Ransil and Schachter, 1994). To provide further insight into the influence of dominance on proprioception, we nevertheless encourage future studies to include established assessments of laterality scores in addition to self-reported global handedness. We also only included completely right-limb dominant individuals for both the upper and lower limbs in the current study. It would be of interest to also assess those with left-limb and mixed-limb dominance and this is recommended for future research.

## Conclusion

Right hemisphere lateralization of brain response during a knee position matching task was evident among right-limb dominant asymptomatic individuals regardless of which limb was active. The inclusion of simultaneous lower limb kinematics during fMRI revealed significantly smaller absolute, but not constant nor variable, errors for the non-dominant left knee when reproducing knee angles compared with the dominant right knee and indicates implications for motor control. Further research should investigate whether similar right brain hemisphere lateralization and non-dominant side preference for proprioception is also evident among left-limb and mixed-limb dominant individuals.

## Data availability statement

The raw data supporting the conclusions of this article will be made available by the authors, without undue reservation.

## Ethics statement

The studies involving human participants were reviewed and approved by Regional Ethical Review Board in Umeå, Sweden. The patients/participants provided their written informed consent to participate in this study.

## Author contributions

CH had the original idea and provided the funding. AS and CH recruited participants. JS created the integrated software used for data collection. AS, AA, and JS performed data collection. AS and HG processed and analyzed the kinematic and brain imaging data. AS, HG, and C-JB performed statistical analyses. AS wrote the first draft of the manuscript. AA, HG, and JS wrote sections of the manuscript. All authors contributed to the conception and design of the study and contributed to manuscript revision, read, and approved the submitted version.
